# Multi‐marker algorithms based on CXCL13, IL‐10, sIL‐2 receptor, and β2‐microglobulin in cerebrospinal fluid to diagnose CNS lymphoma

**DOI:** 10.1002/cam4.3048

**Published:** 2020-04-20

**Authors:** Masahiro Maeyama, Takashi Sasayama, Kazuhiro Tanaka, Satoshi Nakamizo, Hirotomo Tanaka, Masamitsu Nishihara, Yuichi Fujita, Kenji Sekiguchi, Masaaki Kohta, Katsu Mizukawa, Takanori Hirose, Tomoo Itoh, Eiji Kohmura

**Affiliations:** ^1^ Department of Neurosurgery Kobe University Graduate School of Medicine Kobe Japan; ^2^ Department of Neurosurgery Nishi‐Kobe Medical Center Kobe Japan; ^3^ Division of Neurology Kobe University Graduate School of Medicine Kobe Japan; ^4^ Division of Pathology for Regional Communication Kobe University School of Medicine Kobe Japan; ^5^ Department of Diagnostic Pathology Hyogo Cancer Center Akashi Japan; ^6^ Department of Diagnostic Pathology Kobe University Hospital Kobe Japan

**Keywords:** biomarker, central nervous system lymphoma, cerebrospinal fluid, CXCL13, IL‐10

## Abstract

Tumor biopsy is essential for the definitive diagnosis of central nervous system (CNS) lymphoma. However, the biopsy procedure carries the risk of complications such as bleeding, convulsions, and infection. Cerebrospinal fluid (CSF) β2‐microglobulin (β2‐MG), soluble IL‐2 receptor (sIL‐2R), and interleukin‐10 (IL‐10) are known to be useful diagnostic biomarkers for CNS lymphoma. The C‐X‐C motif chemokine ligand 13 (CXCL13) was recently reported to be another useful biomarker for CNS lymphoma. The purpose of this study is to establish a diagnostic algorithm that can avoid biopsy by combining these diagnostic biomarkers. In the first, we conducted a case‐control study (n = 248) demonstrating that the CSF CXCL13 concentration was significantly increased in CNS lymphoma patients compared with various other brain diseases (AUC = 0.981). We established a multi‐marker diagnostic model using CSF CXCL13, IL‐10, β2‐MG, and sIL‐2R from the results of the case‐control study and then applied the model to a prospective study (n = 104) to evaluate its utility. The multi‐marker diagnostic algorithms had excellent diagnostic performance: the sensitivity, specificity, positive predictive value, and negative predictive value were 97%, 97%, 94%, and 99%, respectively. In addition, CSF CXCL13 was a prognostic biomarker for CNS lymphoma patients. Our study suggests that multi‐marker algorithms are important diagnostic tools for patients with CNS lymphoma.

## Graphical abstract

A comparison of the cerebrospinal fluid (CSF) concentrations of CXCL13 between CNS lymphomas and other CNS diseases. The CSF CXCL13 levels of CNS lymphoma were significantly higher than those of the other diseases. The CXCL13 expression levels increased in the CNS lymphoma specimens compared with the other tumor specimens. The multi‐marker prediction algorithms based on CSF CXCL13, IL‐10, sIL‐2R, and β2‐MG had excellent diagnostic performance.

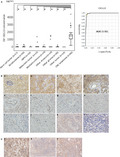

**Abbreviations**
AIC: Akaike information criterion
AUC: area under the ROC curve
BIC: Bayesian information criterion
BLR‐1: Burkitt's lymphoma receptor 1
CSF: cerebrospinal fluid
CXCL13: C‐X‐C motif chemokine ligand 13
CXCR5: C‐X‐C chemokine receptor type 5
GBM: glioblastoma
IL‐10: interleukin‐10
iNPH, idiopathic normal pressure hydeocephalus; MRI: magnetic resonance imaging
MTX: methotrexate
NHL: non‐Hodgkin lymphoma
OS: overall survival
PCNSL: primary central nervous system lymphoma
PFS: progression‐free survival
RMSR: root mean squared error
ROC: receiver operating characteristic
SCNSL: secondary central nervous system lymphoma
sIL‐2R: soluble IL‐2 receptor
β2‐MG: β2‐microglobulin

## 1 INTRODUCTION

Central nervous system lymphoma (CNS lymphoma) is an aggressive extranodal non‐Hodgkin lymphoma (NHL) and is found in approximately 4% of all brain tumors.^1^ With the aging of society and the spread of immunosuppressants and anticancer drugs, the number of patients has increased in the past few decades.^2^ Treatments using high‐dose methotrexate (MTX) have produced acceptable responses in CNS lymphoma patients, and combined modality therapy has led to response rates of 80%‐90%. However, CNS lymphoma has a worse prognosis than other extranodal NHLs.^1^

Tumors frequently occur in the corpus callosum, cerebellum, and in the cerebral white matter near the lateral ventricles.^3^ As the tumors are highly cellular and have decreased water content, magnetic resonance imaging (MRI) T2‐weighted images show shortening and have a relatively low signal intensity and diffusion‐weighted imaging show high signal intensity.^3^ MR spectroscopy of myoinositol may be useful for distinguishing CNS lymphoma from gliomas.^4^ However, CNS lymphomas can simulate other brain diseases, such as metastatic tumor and glioma. Thus, diagnosing CNS lymphoma by radiographic appearance remains challenging.

Tumor biopsy is needed to confirm the diagnosis of CNS lymphoma. However, the biopsy procedure has a certain rate of complications such as bleeding.^5^ Cytology of cerebrospinal fluid (CSF) is a less invasive procedure; however, these are only positive in cases of leptomeningeal involvement. Several useful diagnostic biomarker proteins in the CSF were recently reported to aid in the diagnosis of CNS lymphoma. CSF soluble IL‐2 receptor (sIL‐2R), β2‐microglobulin (β2‐MG), and interleukin‐10 (IL‐10) are known to be useful diagnostic biomarkers.^6^, ^7^, ^8^, ^9^ Rubinstein et al reported good diagnostic accuracy for C‐X‐C motif chemokine ligand 13 (CXCL13) in CSF of CNS lymphoma in a recent large‐scale study.^10^

CXCL13 is strongly expressed in the follicles of the spleen and lymph nodes, and promotes the migration of B‐lymphocytes.^11^ CXCL13 stimulates CXCR5 (C‐X‐C chemokine receptor type 5) expressed in B‐lymphocytes, therefore functions in the homing of B‐lymphocytes to follicles. Three studies analyzed CXCL13 as a marker in CNS lymphoma. A relatively small study (n = 70) showed a significant difference in the CXCL13 levels between CNS lymphoma patients and control.^12^ A relatively large study (n = 220) showed a high specificity of CSF CXCL13 level, and the combination of CXCL13 and IL‐10 is highly useful for the diagnosis of CNS lymphoma.^10^ Another study (n = 87) showed the combined diagnostic performance of CXCL13, IL‐10, and the apparent diffusion coefficient (ADC) on brain MRI.^13^

This study established useful CSF multi‐marker prediction algorithms to diagnose CNS lymphoma. We first evaluated the diagnostic utility of CSF CXCL13 in patients with CNS lymphomas. We then used a logistic regression model to construct multi‐marker prediction algorithms based on four CSF markers of CXCL13, IL‐10, β2‐MG, and sIL‐2R using 143 case‐control study patients. Next, we validated the diagnostic performance of the multi‐marker prediction algorithms applied to the patients in a prospective study. We confirmed that CSF CXCL13 is a useful diagnostic marker for patients with CNS lymphomas. The multi‐marker prediction algorithms based on CSF CXCL13, IL‐10, sIL‐2R, and β2‐MG had excellent diagnostic performance with high sensitivity and specificity. Our study suggests that the CSF multi‐marker prediction algorithm can help diagnose CNS lymphoma with surgical difficulties and be used for treatment planning.

## 2 MATERIALS AND METHODS

### 2.1 Case‐control study (January 2004 ~ December 2015)

To evaluate the diagnostic ability of the CSF CXCL13 levels in patients with CNS lymphomas, 248 patients treated at the Department of Neurosurgery of the Kobe University from January 2004 to December 2015 were retrospectively examined. The patients included 66 CNS lymphoma cases and 182 controls: 66 cases of primary central nervous system lymphoma (PCNSL), 59 cases of glioblastomas (GBMs), 36 cases of other gliomas, 17 cases of germ cell tumors, 13 cases of metastatic brain tumors, 14 cases of other tumors, 14 cases of multiple sclerosis, 10 cases of idiopathic normal pressure hydrocephalus (iNPH), and 19 cases of other brain diseases. Detailed patient data are shown in Table S1. To generate the multi‐marker diagnostic algorithms for CNS lymphoma, IL‐10, sIL‐2R, and β2‐MG in CSF were measured in 143 patients in the case‐control study in addition to CXCL13 (Table S1). All of the CSF samples were preoperatively drawn from the patients by lumbar puncture after informed consent was obtained and stored at −70°C. This study was approved by the ethical review board of our institution (No. 1312).

### 2.2 Prospective study for multi‐marker diagnostic algorithms (January 2016 ~ December 2018)

To verify the diagnostic ability of the multi‐marker predictive algorithms based on the results of the case‐control study, we conducted a prospective study at the Department of Neurosurgery of the Kobe University from January 2016 through December 2018. The patients who were preoperatively suspected of having CNS lymphomas by MRI were included. Patients with symptoms of increasing intracranial pressure were excluded because of a risk of lumber puncture. In addition, patients with severe brain shift because of tumor and/or peritumoral edema were also excluded. A total of 104 patients were enrolled in this study. To examine the CSF samples, the patients underwent lumbar puncture after informed consent was obtained and their CXCL13, IL‐10, β2‐MG, and sIL‐2R levels were preoperatively analyzed in combination with routine biochemical examinations including protein and lactate dehydrogenase. Serum samples were also obtained from 67 patients. For the pathological diagnosis, brain biopsies or tumor removals were performed after CSF examination. This prospective study was approved by the ethical review board of our institution (No. B190031).

### 2.3 Measurement of CXCL13, IL‐10, β2‐MG, and sIL‐2R in CSF and serum

The CSF and blood samples were immediately centrifuged and stored at −70°C. The CSF and serum concentration of CXCL13 were measured using a Human BLC ELISA Kit (ELH‐BLC‐1; RayBiotech Life) and plate reader (Skanlt 3.1 for Multiskan FC; Thermo Fisher Scientific). The CSF concentration of IL‐10 was measured using a human IL‐10 ELISA kit (KHC0101; Life Technologies) and a plate reader (Emax; Molecular Devices at an SRL company (SRL). The CSF concentration of sIL‐2R was measured using a sandwich ELISA test kit (CellfreeN IL‐2R, Kyowa Medex Co., Ltd.) in a fully automated EIA analytical instrument (AP‐X, Kyowa Medex Co., Ltd.) at an SRL company (SRL). The CSF concentration of β2‐MG was measured using a latex agglutination turbidimetric immunoassay. The limits of the tests for the quantification of CXCL13, IL‐10, sIL‐2R, and β2‐MG levels were 1 pg/mL, 2 pg/mL, 50 U/mL, and 200 μg/L, respectively.

### 2.4 Immunohistochemistry of CXCL13 and CXCR5

Antibodies to CXCL13 and CXCR5 were purchased from Boster Biological Technology and Abcam. Archived paraffin blocks from the department of pathology of our hospital were used. The sections were heated in 0.01 mol/L citrate buffer (pH 6.0) for 15 min by autoclaving (121°C, 2 atm).

### 2.5 Survival analyses of the PCNSL patients

There are a total of 93 patients with PCNSL; the case‐control study had 66 patients and the prospective study had 27 patients. For the survival analysis, we selected the newly diagnosed patients with PCNSL treated with chemotherapy and/or radiotherapy. Thirty patients were excluded and 63 patients were analyzed. Of the 63 patients with PCNSL, high‐dose MTX‐based chemotherapy + radiotherapy (RT) was administered to 49 patients. MTX (3‐6 g/m^2^) was administered as an intravenous infusion over 4 hours. MTX‐based chemotherapy was administered for 1‐4 cycles in each patient. In whole brain irradiation, both eyes were included in the RT field, and the radiation dose was typically 36 Gy (1.8 Gy × 20 fractions). The boost irradiation dose for the tumor was 10‐20 Gy. Follow‐up MRI scans were conducted every 3‐6 months or if clinically necessary. Progression‐free survival (PFS) was determined from the onset of treatment until relapse, disease progression, or the last follow‐up evaluation. Overall survival (OS) was determined from the onset of treatment until the last follow‐up evaluation or death from any cause.

### 2.6 Establishment of multi‐marker predictive models

The CSF data from 143 patients examining all four markers (CXCL13, IL‐10, β2‐MG, and sIL‐2R) of the case‐control study were used to establish the multi‐marker predictive algorithms. Logistic regression analysis was applied to generate the diagnostic algorithms and obtain the best sensitivity/specificity to predict disease presence. The coefficient of determination (*R* ^2^) was used to measure how close the data were to the fitted regression line. Akaike information criterion (AIC), the Bayesian information criterion (BIC), and root mean squared error (RMSE) were used as measures of lack of fit (lower values indicated better fit) and the area under the ROC curve (AUC) was used as a discrimination measure. The AIC and BIC penalized model complexity, favoring simpler models, while the RMSE and AUC focused on the predictive ability itself, favoring more complex models. To evaluate the diagnostic ability of the multi‐marker predictive algorithms indicated as best according to the aforementioned indexes (AIC, BIC, RMSE, and AUC), the sensitivity and specificity terms were provided. The logistic regression analyses were conducted using JMP 11 statistical software (JMP Institute). All of the analyses were supported by biostatisticians at the Kobe university clinical and translational research center.

### 2.7 Evaluation of the multi‐marker diagnostic models

To evaluate the multi‐marker diagnostic models obtained from the results of the case‐control study, we fitted them to the prospective study patients. The optimal threshold for discrimination of CNS lymphoma from other diseases was 0.50, meaning that a patient with a higher score was considered to have CNS lymphoma and a patient with a score lower than 0.50 was considered to not have CNS lymphoma. The sensitivity and specificity were calculated in the prospective study patients using this cutoff value of 0.5.

### 2.8 Statistical analysis

The Mann‐Whitney *U*‐test was used to analyze the differences between the two groups. Receiver operating characteristic (ROC) curves were created and the area under the curve (AUC) was calculated to evaluate the diagnostic accuracy. Survival was estimated using Cox's proportional hazards model and the Kaplan‐Meier method, and the significance was determined using the log‐rank and Wilcoxon's test. A *P* < .05 was considered statistically significant. The statistical analysis was conducted using JMP 11 software (JMP Institute). All of the analyses were supported by biostatisticians at the Kobe university clinical and translational research center.

## 3 RESULTS

### 3.1 CSF CXCL13 is a useful biomarker for CNS lymphoma (case‐control study)

**FIGURE 1:**
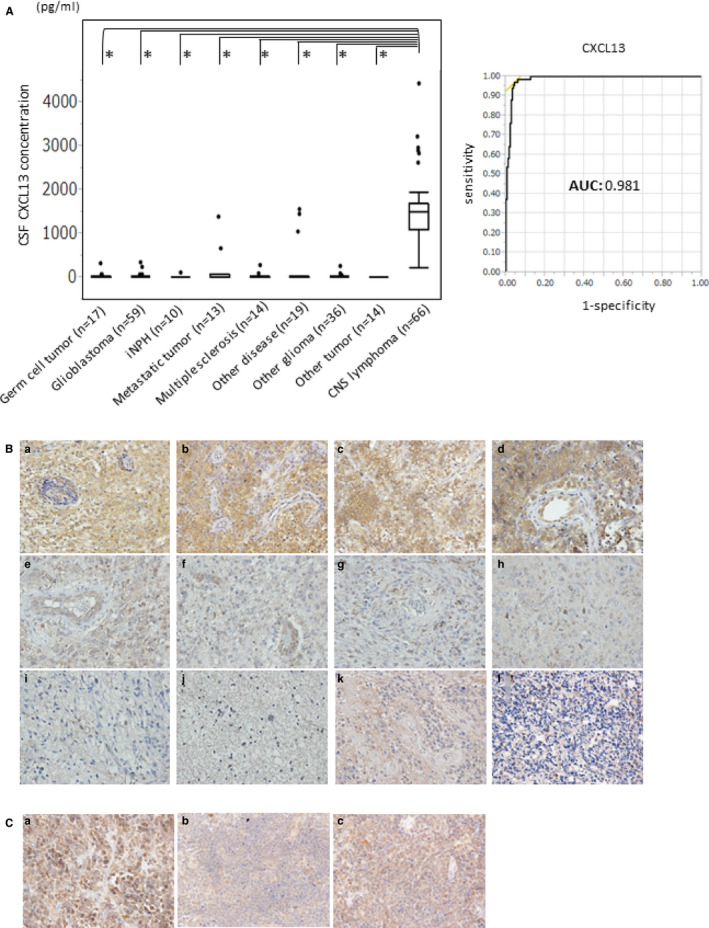
A, A comparison of the cerebrospinal fluid (CSF) concentrations of CXCL13 between CNS lymphomas and other CNS diseases (germ cell tumor, glioblastoma, other glioma, metastatic tumor, other brain tumor, multiple sclerosis, iNPH, and other diseases). The CSF CXCL13 levels of CNS lymphoma were significantly higher than those of the other diseases. (*P < .001). B, ROC curve of CSF CXCL13. AUC is 0.981. C, Immunohistochemistry of CXCL13 in CNS lymphoma specimens and other brain tumor specimens. The CXCL13 expression levels increased in the CNS lymphoma specimens compared with the other tumor specimens. (a‐d) CNS lymphoma, (e‐f) GBM, (g) anaplastic oligodendroglioma, (h) anaplastic astrocytoma, (i‐j) diffuse astrocytoma, (k) ependymoma, and (l) medulloblastoma (original magnification: ×200). D, Immunohistochemistry of CXCR5 in the CNS lymphoma specimens (original magnification: ×200)

The case‐control study analyzed 248 patients. Of these, 66, 59, 36, 17, 13, 14, 14, 10, and 19 had CNS lymphomas, glioblastomas, other gliomas, germ cell tumors, metastatic tumors, other tumors, multiple sclerosis, iNPH, and other diseases, respectively. The mean concentrations of CSF CXCL13 in CNS lymphoma and non‐CNS lymphoma were 1483 ± 731 and 55 ± 209 pg/mL, respectively. The mean concentrations of CSF CXCL13 in glioblastomas, other gliomas, germ cell tumors, metastatic tumors, other tumors, multiple sclerosis, iNPH, and other diseases were 21 ± 214, 24 ± 45, 38 ± 77, 176 ± 390, 4 ± 3, 36 ± 72, 14 ± 31, and 233 ± 498 pg/mL, respectively. The CSF CXCL13 levels in CNS lymphoma were significantly higher than in the other CNS diseases (Figure 1A). The ROC curve of the CSF CXCL13 levels for CNS lymphoma showed high sensitivity and specificity (AUC = 0.981; Figure 1B). However, four cases of other diseases had high CSF CXCL13 over 1000 pg/mL, IgG4‐related disease: 1568 and 1043 pg/mL, sarcoidosis: 1390 pg/mL, and metastatic brain tumor: 1320 pg/mL (Figure 1A; Table S1). Two patients with IgG4‐related disease had inflammation of the pituitary gland and pituitary stalk without pachymeningitis. In the sarcoidosis patient, multiple contrast‐enhanced lesions were found on the temporal lobe, lateral ventricle wall, and fourth ventricular wall. In the patient with metastatic brain tumor from lung, the tumor was found in the basal ganglia.

### 3.2 CXCL13 and CXCR5 were expressed in CNS lymphoma cells

To examine the CXCL13 expression, immunohistochemical examination was conducted using the case‐control study specimens. As shown in Figure 1C, CXCL13 protein was highly expressed in the CNS lymphoma cells (Figure 1C, a‐d). Other tumors, such as glioblastoma, anaplastic oligodendroglioma, anaplastic astrocytoma, diffuse astrocytoma, ependymoma, and medulloblastoma, had a lower expression of CXCL13 protein (Figure 1C, e‐l). CXCR5, a receptor of CXCL13, was highly expressed in the CNS lymphomas (Figure 1D). These results indicate that the CXCL13/CXCR5 signaling pathway may be activated in CNS lymphoma cells.

### 3.3 Establishment of multi‐marker diagnostic algorithms for CNS lymphoma

In addition with CSF CXCL13, we examined the CSF concentrations of IL‐10, β2‐MG, and sIL‐2R in 143 patients in the case‐control study (Figure S1; Table S1). To construct the multi‐marker prediction algorithms, we applied the logistic regression models to the CSF CXCL13, IL‐10, β2‐MG, and sIL‐2R data. (Table 1). The 2‐marker model of CXCL13 + IL‐10 had the lowest AIC and BIC, while the 4‐marker model of CXCL13 + IL‐10 + β2‐MG + sIL‐2R had the lowest RMSE and highest AUC. The 3‐marker model of CXCL13 + IL‐10 + β2‐MG or sIL‐2R also had very low AIC, BIC, and RMSE and very high AUC. We evaluated four multi‐marker diagnostic models for their diagnoses of CNS lymphoma. Their formulas follow:

CNS lymphoma probability: *P* = 1/1 + Exp(−*Z*)
2‐marker model (CXCL13 + IL‐10): *Z* = −9.26 + CXCL13 × (0.0037) + IL‐10 × (1.87)
3‐marker model 1 (CXCL13 + IL‐10 + β2‐MG): *Z* = −10.72 + CXCL13 × (0.0038) + IL‐10 × (1.69) + β2‐MG × (0.0011)
3‐marker model 2 (CXCL13 + IL‐10 + sIL‐2R): *Z* = −9.30 + CXCL13 × (0.0037) + IL‐10 × (1.87) + sIL‐2R × (0.00062)
4‐marker model (CXCL13 + IL‐10 + β2‐MG + sIL‐2R): *Z* = −10.79 + CXCL13 × (0.0039) + IL‐10 × (1.71) + β2‐MG × (0.0013) + sIL‐2R × (−0.0052)

**TABLE 1:** Performance of each model possibility by case‐control study

Markers included in the model	Performance index					
	AIC	BIC	RMSE	AUC	*R* ^2^	*P*‐value
CXCL13	64.4	70.2	0.236	0.979	.682	<.0001
IL‐10	41.6	47.4	0.185	0.971	.802	<.0001
β2‐MG	91.0	96.8	0.297	0.927	.542	<.0001
sIL‐2R	149.0	154.8	0.398	0.845	.236	<.0001
CXCL13 + IL‐10	**23.6**	**32.3**	0.137	**0.998**	.908	<.0001
CXCL13 + β2‐MG	41.8	50.5	0.190	0.990	.812	<.0001
CXCL13 + sIL‐2R	59.1	67.8	0.230	0.984	.721	<.0001
IL‐10 + β2‐MG	38.0	46.7	0.163	0.978	.832	<.0001
IL‐10 + sIL‐2R	43.5	52.2	0.186	0.978	.803	<.0001
β2‐MG + sIL‐2R	90.1	98.8	0.289	0.935	.557	<.0001
CXCL13 + IL‐10 + β2‐MG	24.1	35.6	0.135	**0.998**	.917	<.0001
CXCL13 + IL‐10 + sIL‐2R	25.7	37.2	0.137	**0.998**	.908	<.0001
CXCL13 + β2‐MG + sIL‐2R	43.8	55.4	0.191	0.990	.813	<.0001
IL‐10 + β2‐MG + sIL‐2R	40.1	51.6	0.162	0.978	.832	<.0001
CXCL13 + IL‐10 + β2‐MG + sIL‐2R	25.9	40.3	**0.134**	**0.998**	**.919**	<.0001

Bold indicates highest or lowest value.

Abbreviations: AIC, Akaike information criterion; AUC, the area under the ROC curve; BIC, Bayesian information criterion; *R* ^2^, *R*‐squared; RMSE, root mean squared error.

### 3.4 Diagnostic performance of the multi‐marker diagnostic models in the prospective study

**FIGURE 2:**
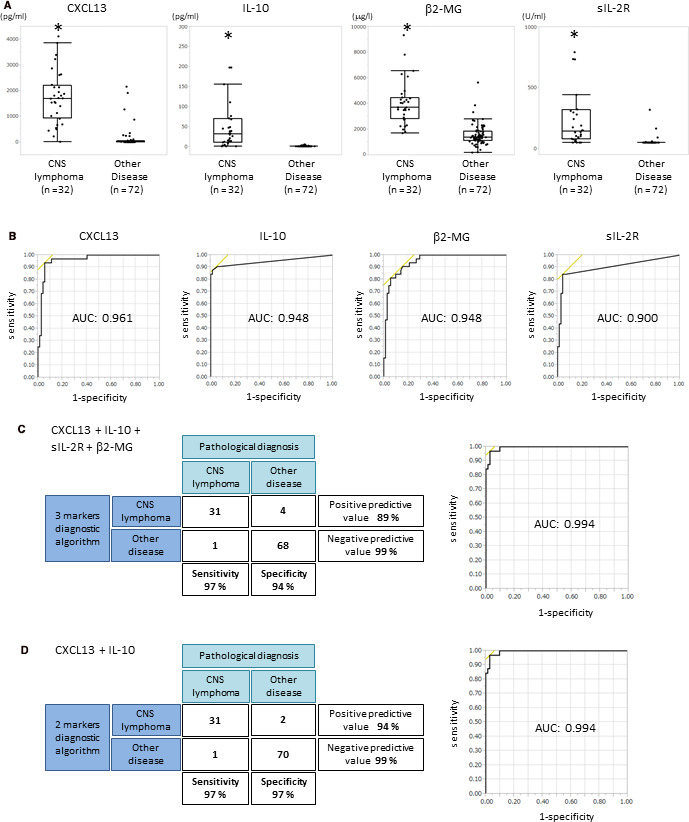
A, A comparison of the cerebrospinal fluid (CSF) concentrations of CXCL13, IL‐10, sIL‐2R, and β2‐MG in the prospective study. Patient data are shown in Table S2. The CSF levels of all biomarkers significantly increased in CNS lymphoma compared with the other diseases (*P < .001). B, ROC curve of CSF CXCL13, IL‐10, sIL‐2R, and β2‐MG in the prospective study. ROC is statistically significant (*P* < .001). AUC values are shown in each graph. C, Correlation of diagnosis by the 4‐marker algorithm of CXCL13, IL‐10, sIL‐2R, and β2‐MG with pathological diagnosis in the prospective study. Sensitivity, specificity, positive predictive value, and negative predictive value are shown in the panels. The ROC curve of these combinations in the prospective study is shown in the right panel. D, Correlation of diagnosis by the 2‐marker algorithm of CXCL13 and IL‐10 with pathological diagnosis in the prospective study. Sensitivity, specificity, positive predictive value, and negative predictive value are shown in the panels. The ROC curve of these combinations in the prospective study is shown in the right panel

We conducted the prospective study of 104 patients to evaluate the aforementioned multi‐marker diagnostic models. The patients’ pathological diagnoses and CSF data are listed in Table S2. Twenty‐seven patients had PCNSL, three had secondary CNS lymphoma (SCNSL), one had B‐lymphoblastic leukemia/lymphoma, one had intravascular lymphoma, and 72 had other diseases. All patients with SCNSL showed relapse in the brain, who had no other lesion except for brain. The CSF levels of all of the markers were significantly increased in the patients with CNS lymphoma (Figure 2A,B). The multi‐marker diagnostic algorithms calculated using the case‐control study were applied to the prospective study patients and the results are shown in Table 2 and Figure 2C,D. The sensitivity and specificity of the algorithms were very high. The diagnostic model of CXCL13 + IL‐10 + sIL‐2R or CXCL13 + IL‐10 was the best (Table 2; Figure 2D). The false positive cases of the 3‐marker (CXCL13, IL‐10, and sIL‐2R) and 2‐marker (CXCL13 and IL‐10) algorithms were due to two cases of histiocytic sarcoma and metastatic brain tumor. Additionally, there were two false positive cases of GBM and brain involvement of Sjögren's syndrome in the 4‐marker (CXCL13, IL‐10, β2‐MG, and sIL‐2R) and 3‐marker (CXCL13, IL‐10, and β2‐MG) algorithms. Interestingly, the patient with Sjögren's syndrome had a past history of gastric MALT lymphoma 10 years prior.

**TABLE 2:** Diagnostic performances of multi‐marker algorithm

	Marker	Sensitivity	Specificity	Positive predictive value	Negative predictive value
4 markers	CXCL13, IL‐10, β2‐M, sIL‐2R	97	94	89	99
3 markers	CXCL13, IL‐10, β2‐M	97	94	89	99
3 markers	CXCL13, IL‐10, sIL‐2R	97	97	94	99
2 markers	CXCL13, IL‐10	97	97	94	99

### 3.5 Serum CXCL13 is not a useful marker for CNS lymphoma

**FIGURE 3:**
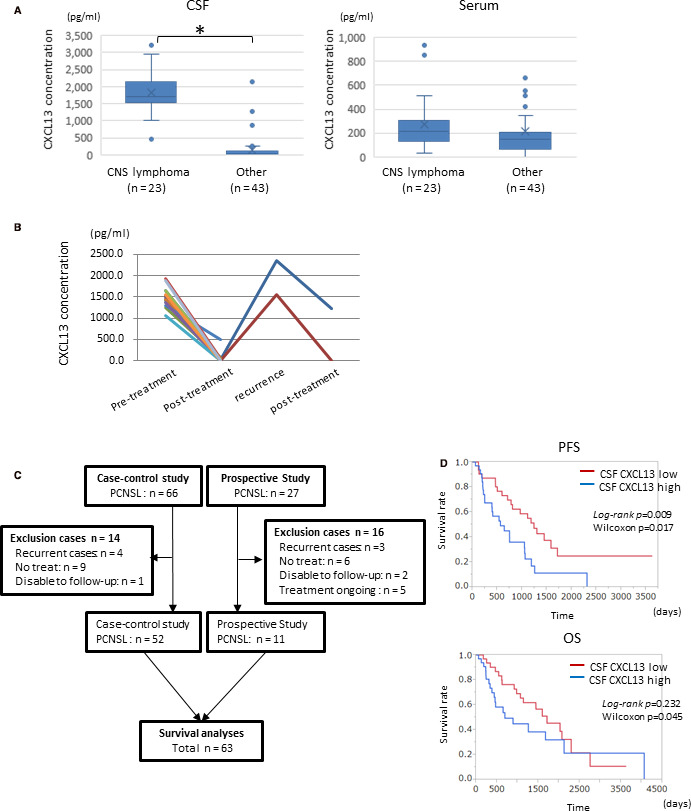
A, Comparison of the serum or cerebrospinal fluid (CSF) levels of CXCL13 between CNS lymphoma and the other CNS diseases. B, Changes in the CSF CXCL13 concentration in the disease process. At recurrence, CSF CXCL13 levels increased again. C, A flow chart of the PCNSL patient selection in the survival analysis. D, Comparison of Kaplan‐Meier PFS (left panel) and OS (right panel) curves according to the concentrations of CSF CXCL13 (high > 1500 pg/mL, low < 1500 pg/mL; p: log‐rank and Wilcoxon analysis)

Of the 104 patients in the prospective study, the serum concentrations of CXCL13 were measured in 67 (Figure 3A). The mean concentrations of serum CXCL13 in CNS lymphoma and other diseases were 269 pg/mL (range 30‐938 pg/mL) and 217 pg/mL (range 1‐2070 pg/mL), respectively. The serum CXCL13 levels of CNS lymphomas were not statistically increased compared with the other diseases, although the CSF CXCL13 levels were significantly increased in CNS lymphoma (Figure 3A). These results indicated that the serum concentration of CXCL13 is not a useful diagnostic biomarker for CNS lymphoma.

### 3.6 Posttreatment CSF CXCL13 levels

In the 14 patients with CNS lymphomas, the CSF concentrations of CXCL13 were measured after the completion of therapy (Figure 3B). The CSF concentration of CXCL13 post‐therapy was significantly decreased compared with pre‐therapy in all of the patients analyzed. Two patients had re‐elevation of CSF CXCL13 levels at the time of relapse, and after the therapy, the CSF CXCL13 levels were decreased again (Figure 3B). These results indicated that CSF CXCL13 is a useful disease monitoring marker for CNS lymphoma.

### 3.7 Relationship between CSF CXCL13 and prognosis

We analyzed the prognosis of the 63 PCNSL patients in the case‐control and prospective studies. Patient selection is shown in Figure 3C and the patients’ clinical characteristics are shown in Table S3. We divided the patients into two groups according to the median value: a high CXCL13 group (> 1500 pg/mL, n = 32) and low CXCL13 group (<1500 pg/mL, n = 31). As shown in Figure 3D, the high CSF CXCL13 group had significantly shorter PFS times (log‐rank *P* = .009, Wilcoxon: *P* = .017) and shorter OS (log‐rank *P* = .232, Wilcoxon *P* = .045; Figure 3D). The multivariate analysis of CXCL13, age, sex, and Karnofsky performance status (KPS) revealed that higher CSF CXCL13 was significantly associated with decreased OS (*P* = .0047, HR = 1.001, 95% CI: 1.000‐1.001; Table 3).

**TABLE 3:** Univariate and multivariate overall survival analyses of PCNSL (n = 63)

	Univariate			Multivariate		
	HR	95% CI	*P*‐value	HR	95% CI	*P*‐value
CSF CXCL13^a^	1.001	1.000 ~ 1.001	.0035	1.001	1.000 ~ 1.001	.0047
Age^a^	1.021	0.985 ~ 1.060	.251	1.018	0.979 ~ 1.058	.357
Sex	1.022	0.501 ~ 1.997	.950	1.028	0.518 ~ 2.128	.938
KPS^a^	1.985	0.957 ~ 1.013	.302	0.992	0.962 ~ 1.022	.606

Abbreviations: CI, confidence interval; HR, hazard ratio; KPS, Karnofsky performance status; PCNSL, primary central nervous system lymphoma.

^a^ Continuous variable.

## 4 DISCUSSION

Clinical and radiological characteristics alone make it difficult to diagnose CNS lymphoma. With imaging alone, such as MRI, demyelinating diseases, inflammatory diseases, and malignancies, such as glioblastoma and metastatic tumors, can be mistakenly diagnosed as CNS lymphoma. Tumor biopsy is required for a definitive diagnosis; however, the biopsy procedure has a risk of complications, such as hemorrhage and seizure. Therefore, it is desired to establish a diagnostic method capable of avoiding tumor biopsy and enabling early diagnosis.

The CSF levels of the following biomarkers have been shown to be elevated in CNS lymphoma: sCD27,^14^, ^15^ sCD19,^16^ β2‐MG,^17^ antithrombin III,^18^ free light‐chain immunoglobulins,^19^ a specific set of microRNAs,^20^, ^21^ IL‐10 ^9^, ^22^ sIL2‐R,^9^ CXCL13,^10^, ^23^ and neopterin.^24^ The diagnostic utility of CSF IL‐10, β2‐MG, and sIL‐2R was also reported in other studies including our previous study.^22^, ^25^, ^26^ However, CSF CXCL13 is a recently discovered diagnostic biomarker for CNS lymphoma.^12^ In the present study, the CSF CXCL13 concentration was significantly increased in the patients with CNS lymphoma. However, CSF CXCL13 is known to be increased in other diseases such as Lyme neuroborreliosis^27^, ^28^, ^29^, ^30^, ^31^ and neurosyphilis.^32^, ^33^ Patients with early stage anti‐NMDAR (N‐methyl‐D‐aspartate receptor) encephalitis had an increased CSF CXCL13 concentration.^34^ Additionally, the CSF CXCL13 concentration was elevated in patients with aseptic meningoencephalitis and neuromyelitis optica (NMO).^35^, ^36^ In our case‐control study, CXCL13 was elevated in four cases in non‐CNS lymphoma. Two cases were IgG4‐RD, one case was sarcoidosis, and one case was metastatic tumor. It has been reported that infiltrated T cells produce CXCL13 in IgG4‐RD,^37^ so that CXCL13 concentration in CSF may increase in IgG4‐RD patients. Sarcoidosis was high in one of four cases, but there were few reports of a relationship between sarcoidosis and CXCL13. However, CXCL13 was elevated in the aqueous humor of uveitis including sarcoidosis.^38^ Two of 18 metastatic brain tumors in our study had high CXCL13 levels. There are several reports of CXCL13 expression in lung cancer. In addition, CXCL13 levels were increased in bronchoalveolar lavage fluid in non‐small cell lung cancer, and high CXCL13 was reported to be a poor prognostic factor.^39^ Immunostaining of metastatic brain tumor tissue with high levels of CSF CXCL13 revealed overexpression of CXCL13 in cancer cells, suggesting that CXCL13 expression was enhanced by some mechanism, but the details are unknown. Since some diseases have high CSF CXCL13 concentrations, diagnosis with a multi‐marker is necessary.

Rubenstein et al previously reported on the usefulness of CNS lymphoma diagnosis with the combination of CXCL13 and IL‐10.^10^ They analyzed 220 cases, including 83 CNS lymphoma, retrospectively, but we analyzed 248 cases retrospectively and 104 cases prospectively, which are a larger number of cases. Also, our statistical analysis is more detailed than theirs, and is novel in that we have constructed an algorithm for diagnosis. Rubenstein et al reported an AUC value of 0.746 for the combination of CXCL13 and IL‐10, which was considerably lower than our AUC value. The reason for the markedly better AUC in our analysis is not clear, but one reason may be related to the administration of steroids. We have confirmed that administration of steroids decreases CSF CXCL13 and IL‐10 concentrations, therefore steroid administration reduces diagnostic accuracy.

This study evaluated multi‐marker diagnostic algorithms with various combinations of CXCL13, IL‐10, β2‐MG, and sIL‐2R. The combination of four markers had a very high discrimination power for CNS lymphoma. The 2‐marker diagnostic model of CXCL13 and IL‐10 and the 3‐marker model of CXCL13, IL‐10 and sIL‐2R also had very high sensitivity and specificity. The 2‐marker model is simplest, but had good AIC and BIC values, and was comparable to the 4‐marker model in diagnostic accuracy; the 2‐marker model had a higher positive predictive value in the prospective study, but when the model equation was applied to patients in the case‐control study, the 2‐marker model had a lower negative predictive value (98%) than the 4‐marker model (99%). In the present study, it was not able to conclude which of the 2‐marker model or the 4‐marker model has the higher diagnostic accuracy; however, the 4‐marker model may be more sensitive and specific to various diseases and steroid administration, and should be verified in many cases in the future.

Given that CNS lymphoma cells express CXCR5, it is tempting to speculate about the role of CXCL13 in the pathogenesis of CNS lymphoma. The CXCL13/CXCR5 signal is associated with mitogen‐activated protein kinase (MAPK) pathway, which participates in lymphocyte activation.^40^ In addition, CXCL13/CXCR5 axis activates the phosphatidylinositol‐3 kinase (PI3K)/AKT pathway, leading to invasion and migration of cancer cells.^41^ Our study demonstrated that CNS lymphoma patients with high CSF CXCL13 levels had poorer OS and PFS. Rubinstein et al reported similar results. In addition, the CXCL13 level is associated with the prognosis in extranodal natural killer (NK)/T‐cell lymphoma patients.^42^

Recently, several reports have shown that circulating tumor DNA (ctDNA) is useful for the diagnosis of CNS lymphoma. MYD88 and CD79B mutations were detected in 38% ~ 76% and 30% ~ 83% of patients with PCNSL, respectively.^43^, ^44^, ^45^ Several mutations can be detected from ctDNA in CSF of PCNSL patients, and MYD88 mutations were detected from CSF at a high rate.^46^ Therefore, multi‐modality diagnostic algorithms including CSF cytokines/chemokines and ctDNA may be established in the future.

Our study has several limitations. First, the size was relatively small, and it was conducted at only one institution. In particular, the number of neuroinflammatory disease cases was small. Second, in prognosis analysis, individual treatment did not follow the same protocol. Most of the patients received both RT and chemotherapy; however, some patients received RT or chemotherapy alone. In addition, several patients were treated with corticosteroids before CSF sampling. The effects of corticosteroids are unknown; however, the CSF concentrations of CSF CXCL13, IL‐10, β2‐MG, and sIL‐2R seemed to be decreased by corticosteroids. Nevertheless, our prospective study demonstrated the diagnostic utility of the CSF multi‐marker predictive algorithms.

In conclusion, CSF CXCL13 is a useful diagnostic biomarker for patients with CNS lymphomas. The CSF CXCL13 level is a useful prognostic factor for patients with CNS lymphoma. In addition, we found that the combined use of CSF CXCL13, IL‐10, sIL‐2R, and β2‐MG demonstrated excellent diagnostic performance. In order for our diagnostic algorithms to be usable in the clinical setting, they need to be validated in large prospective cohort studies. When the diagnostic value of these combinations of CSF markers is confirmed, risk of biopsy could be avoided and treatment can be started earlier for patients with CNS lymphoma.

## CONFLICT OF INTEREST

All authors have no conflict of interest to declare.

## AUTHOR CONTRIBUTIONS

TS conceived this work, performed CSF/serum analyses, and wrote the paper. MM performed CSF/serum analyses and wrote the paper. KT and MK performed statistical analyses. SN, HT, MN, YF, KS, and MK collected and stored the CSF/serum samples. TH and TI performed pathological diagnosis. EK supervised the whole study.

## ETHICAL STATEMENT

This study was approved by the ethical review board of our institutions (No. 1312) (No. B190031).

## Supporting information

Figure S1

Table S1

Table S2

Table S3

## Data Availability

The data that support the findings of this study are available from the corresponding author upon reasonable request.

## References

[1] Olson JE , Janney CA , Rao RD , et al. The continuing increase in the incidence of primary central nervous system non‐Hodgkin lymphoma: a surveillance, epidemiology, and end results analysis. Cancer. 2002;95:1504‐1510. 12237919 10.1002/cncr.10851

[2] Sierra del Rio M , Rousseau A , Soussain C , Ricard D , Hoang‐Xuan K . Primary CNS lymphoma in immunocompetent patients. Oncologist. 2009;14:526‐539. 19433528 10.1634/theoncologist.2008-0236

[3] Bataille B , Delwail V , Menet E , et al. Primary intracerebral malignant lymphoma: report of 248 cases. J Neurosurg. 2000;92:261‐266. 10659013 10.3171/jns.2000.92.2.0261

[4] Nagashima H , Sasayama T , Tanaka K , et al. Myo‐inositol concentration in MR spectroscopy for differentiating high grade glioma from primary central nervous system lymphoma. J Neurooncol. 2018;136:317‐326. 29143277 10.1007/s11060-017-2655-x

[5] Khatab S , Spliet W , Woerdeman PA . Frameless image‐guided stereotactic brain biopsies: emphasis on diagnostic yield. Acta Neurochir (Wien). 2014;156:1441‐1450. 24898761 10.1007/s00701-014-2145-2

[6] Jeffery GM , Frampton CM , Legge HM , Hart DN . Cerebrospinal fluid B2‐microglobulin levels in meningeal involvement by malignancy. Pathology. 1990;22:20‐23. 2194155 10.3109/00313029009061421

[7] Chang CS , Liu HW , Lin SF , Chen TP . Soluble interleukin‐2 receptor levels in cerebrospinal fluid of patients with acute lymphocytic leukemia or with non‐Hodgkin's lymphoma. Zhonghua Min Guo Wei Sheng Wu Ji Mian Yi Xue Za Zhi. 1989;22:132‐137. 2605973

[8] Lee W , Kim SJ , Lee S , et al. Significance of cerebrospinal fluid sIL‐2R level as a marker of CNS involvement in acute lymphoblastic leukemia. Ann Clin Lab Sci. 2005;35:407‐412. 16254256

[9] Sasayama T , Nakamizo S , Nishihara M , et al. Cerebrospinal fluid interleukin‐10 is a potentially useful biomarker in immunocompetent primary central nervous system lymphoma (PCNSL). Neuro Oncol. 2012;14:368‐380. 22156547 10.1093/neuonc/nor203 PMC3280797

[10] Rubenstein JL , Wong VS , Kadoch C , et al. CXCL13 plus interleukin 10 is highly specific for the diagnosis of CNS lymphoma. Blood. 2013;121:4740‐4748. 23570798 10.1182/blood-2013-01-476333 PMC3674672

[11] Ansel KM , Harris RB , Cyster JG . CXCL13 is required for B1 cell homing, natural antibody production, and body cavity immunity. Immunity. 2002;16:67‐76. 11825566 10.1016/s1074-7613(01)00257-6

[12] Fischer L , Korfel A , Pfeiffer S , et al. CXCL13 and CXCL12 in central nervous system lymphoma patients. Clin Cancer Res. 2009;15:5968‐5973. 19773382 10.1158/1078-0432.CCR-09-0108

[13] Mabray MC , Barajas RF , Villanueva‐Meyer JE , et al. The combined performance of ADC, CSF CXC chemokine ligand 13, and CSF interleukin 10 in the diagnosis of central nervous system lymphoma. Am J Neuroradiol. 2016;37:74‐79. 26381553 10.3174/ajnr.A4450 PMC4713285

[14] van den Bent MJ , Lamers CH , van't Veer MB , Sillevis Smitt PA , Bolhuis RL , Gratama JW . Increased levels of soluble CD27 in the cerebrospinal fluid are not diagnostic for leptomeningeal involvement by lymphoid malignancies. Ann Hematol. 2002;81:187‐191. 11976819 10.1007/s00277-002-0448-5

[15] Kersten MJ , Evers LM , Dellemijn PL , et al. Elevation of cerebrospinal fluid soluble CD27 levels in patients with meningeal localization of lymphoid malignancies. Blood. 1996;87:1985‐1989. 8634448

[16] Muñiz C , Martín‐Martín L , López A , et al. Contribution of cerebrospinal fluid sCD19 levels to the detection of CNS lymphoma and its impact on disease outcome. Blood. 2014;123:1864‐1869. 24501214 10.1182/blood-2013-11-537993

[17] Caudie C , Bancel J , Dupont M , Matanza D , Poitevin F , Honnorat J . CSF levels and diagnostic utility of cerebrospinal fluid beta2‐microglobulin. Ann Biol Clin (Paris). 2005;63:631‐637. 16330382

[18] Roy S , Josephson SA , Fridlyand J , et al. Protein biomarker identification in the CSF of patients with CNS lymphoma. J Clin Oncol. 2008;26:96‐105. 18056677 10.1200/JCO.2007.12.1053 PMC4134101

[19] Schroers R , Baraniskin A , Heute C , et al. Detection of free immunoglobulin light chains in cerebrospinal fluids of patients with central nervous system lymphomas. Eur J Haematol. 2010;85:236‐242. 20528903 10.1111/j.1600-0609.2010.01475.x

[20] Baraniskin A , Kuhnhenn J , Schlegel U , Schmiegel W , Hahn S , Schroers R . MicroRNAs in cerebrospinal fluid as biomarker for disease course monitoring in primary central nervous system lymphoma. J Neurooncol. 2012;109:239‐244. 22729947 10.1007/s11060-012-0908-2

[21] Baraniskin A , Kuhnhenn J , Schlegel U , et al. Identification of microRNAs in the cerebrospinal fluid as marker for primary diffuse large B‐cell lymphoma of the central nervous system. Blood. 2011;117:3140‐3146. 21200023 10.1182/blood-2010-09-308684

[22] Sasagawa Y , Akai T , Tachibana O , Iizuka H . Diagnostic value of interleukin‐10 in cerebrospinal fluid for diffuse large B‐cell lymphoma of the central nervous system. J Neurooncol. 2015;121:177‐183. 25258254 10.1007/s11060-014-1622-z

[23] Krumbholz M , Theil D , Cepok S , et al. Chemokines in multiple sclerosis: CXCL12 and CXCL13 up‐regulation is differentially linked to CNS immune cell recruitment. Brain. 2006;129:200‐211. 16280350 10.1093/brain/awh680

[24] Viaccoz A , Ducray F , Tholance Y , et al. CSF neopterin level as a diagnostic marker in primary central nervous system lymphoma. Neuro Oncol. 2015;17(11):1497‐1503. 26014047 10.1093/neuonc/nov092 PMC4648303

[25] Hashiguchi S , Momoo T , Murohashi Y , et al. Interleukin 10 Level in the cerebrospinal fluid as a possible biomarker for Lymphomatosis Cerebri. Intern Med. 2015;54:1547‐1552. 26073248 10.2169/internalmedicine.54.3283

[26] Ikeguchi R , Shimizu Y , Shimizu S , Kitagawa K . CSF and clinical data are useful in differentiating CNS inflammatory demyelinating disease from CNS lymphoma. Mult Scler. 2018;24:1212‐1223. 28657431 10.1177/1352458517717804

[27] Yang J , Han X , Liu A , et al. Chemokine CXC ligand 13 in cerebrospinal fluid can be used as an early diagnostic biomarker for Lyme neuroborreliosis: a meta‐analysis. J Interferon Cytokine Res. 2017;37:433‐439. 28972436 10.1089/jir.2016.0101

[28] Hytonen J , Kortela E , Waris M , Puustinen J , Salo J , Oksi J . CXCL13 and neopterin concentrations in cerebrospinal fluid of patients with Lyme neuroborreliosis and other diseases that cause neuroinflammation. J Neuroinflammation. 2014;11:103. 24920219 10.1186/1742-2094-11-103 PMC4070086

[29] Kingwell K . Infectious disease: CXCL13 is a potential biomarker for Lyme neuroborreliosis. Nat Rev Neurol. 2011;7:244. 10.1038/nrneurol.2011.50 21487424

[30] Schmidt C , Plate A , Angele B , et al. A prospective study on the role of CXCL13 in Lyme neuroborreliosis. Neurology. 2011;76:1051‐1058. 21422457 10.1212/WNL.0b013e318211c39a

[31] Remy MM , Schobi N , Kottanattu L , Pfister S , Duppenthaler A , Suter‐Riniker F . Cerebrospinal fluid CXCL13 as a diagnostic marker of neuroborreliosis in children: a retrospective case‐control study. J Neuroinflammation. 2017;14:173. 28859668 10.1186/s12974-017-0948-9 PMC5580331

[32] Wang C , Wu K , Yu Q , et al. CXCL13, CXCL10 and CXCL8 as potential biomarkers for the diagnosis of neurosyphilis patients. Sci Rep. 2016;6:33569. 27650493 10.1038/srep33569 PMC5030708

[33] Dersch R , Hottenrott T , Senel M , et al. The chemokine CXCL13 is elevated in the cerebrospinal fluid of patients with neurosyphilis. Fluids Barriers CNS. 2015;12:12. 25975424 10.1186/s12987-015-0008-8 PMC4489031

[34] Leypoldt F , Hoftberger R , Titulaer MJ , et al. Investigations on CXCL13 in anti‐N‐methyl‐D‐aspartate receptor encephalitis: a potential biomarker of treatment response. JAMA Neurol. 2015;72:180‐186. 25436993 10.1001/jamaneurol.2014.2956 PMC4836910

[35] Fujimori J , Nakashima I , Kuroda H , Fujihara K , Aoki M . Cerebrospinal fluid CXCL13 is a prognostic marker for aseptic meningitis. J Neuroimmunol. 2014;273:77‐84. 24907903 10.1016/j.jneuroim.2014.05.008

[36] Zhong X , Wang H , Dai Y , et al. Cerebrospinal fluid levels of CXCL13 are elevated in neuromyelitis optica. J Neuroimmunol. 2011;240–241:104‐108. 10.1016/j.jneuroim.2011.10.001 22036953

[37] Kamekura R , Takahashi H , Ichimiya S . New insights into IgG4‐related disease: emerging new CD4+ T‐cell subsets. Curr Opin Rheumatol. 2019;31:9‐15. 30422824 10.1097/BOR.0000000000000558 PMC6254779

[38] El‐Asrar AMA , Berghmans N , Al‐Obeidan SA , et al. Differential CXC and CX3C chemokine expression profiles in aqueous humor of patients with specific endogenous uveitic entities. Invest Ophthalmol Vis Sci. 2018;59:2222‐2228. 29715366 10.1167/iovs.17-23225

[39] Naumnik W , Panek B , Ossolinska M , Naumnik B . B cell‐attracting chemokine‐1 and progranulin in bronchoalveolar lavage fluid of patients with advanced non‐small cell lung cancer: new prognostic factors. Adv Exp Med Biol. 2019;1150:11‐16. 30357709 10.1007/5584_2018_285

[40] Muller G , Lipp M . Signal transduction by the chemokine receptor CXCR5: structural requirements for G protein activation analyzed by chimeric CXCR1/CXCR5 molecules. Biol Chem. 2001;382:1387‐1397. 11688722 10.1515/BC.2001.171

[41] Zhu Z , Zhang X , Guo H , Fu L , Pan G , Sun Y . CXCL13‐CXCR5 axis promotes the growth and invasion of colon cancer cells via PI3K/AKT pathway. Mol Cell Biochem. 2015;400:287‐295. 25476740 10.1007/s11010-014-2285-y

[42] Kim SJ , Ryu KJ , Hong M , Ko YH , Kim WS . The serum CXCL13 level is associated with the Glasgow Prognostic Score in extranodal NK/T‐cell lymphoma patients. J Hematol Oncol. 2015;8:49. 25966773 10.1186/s13045-015-0142-4 PMC4437674

[43] Nakamura T , Tateishi K , Niwa T , et al. Recurrent mutations of CD79B and MYD88 are the hallmark of primary central nervous system lymphomas. Neuropathol Appl Neurobiol. 2016;42:279‐290. 26111727 10.1111/nan.12259

[44] Braggio E , Van Wier S , Ojha J , et al. Genome‐wide analysis uncovers novel recurrent alterations in primary central nervous system lymphomas. Clin Cancer Res. 2015;21:3986‐3994. 25991819 10.1158/1078-0432.CCR-14-2116 PMC4558226

[45] Bruno A , Boisselier B , Labreche K , et al. Mutational analysis of primary central nervous system lymphoma. Oncotarget. 2014;5:5065‐5075. 24970810 10.18632/oncotarget.2080 PMC4148122

[46] Watanabe J , Natsumeda M , Kanemaru Y , et al. Comparison of circulating tumor DNA between body fluids in patients with primary central nervous system lymphoma. Leuk Lymphoma. 2019;60:3587‐3589. 31305194 10.1080/10428194.2019.1639169

