# Supplementary figures and images for: Multi‐marker algorithms based on CXCL13, IL‐10, sIL‐2 receptor, and β2‐microglobulin in cerebrospinal fluid to diagnose CNS lymphoma
Source: Cancer Med. 2020 Apr 20;9(12):4114–25. doi: 10.1002/cam4.3048 (PMC7300423; doi:10.1002/cam4.3048)

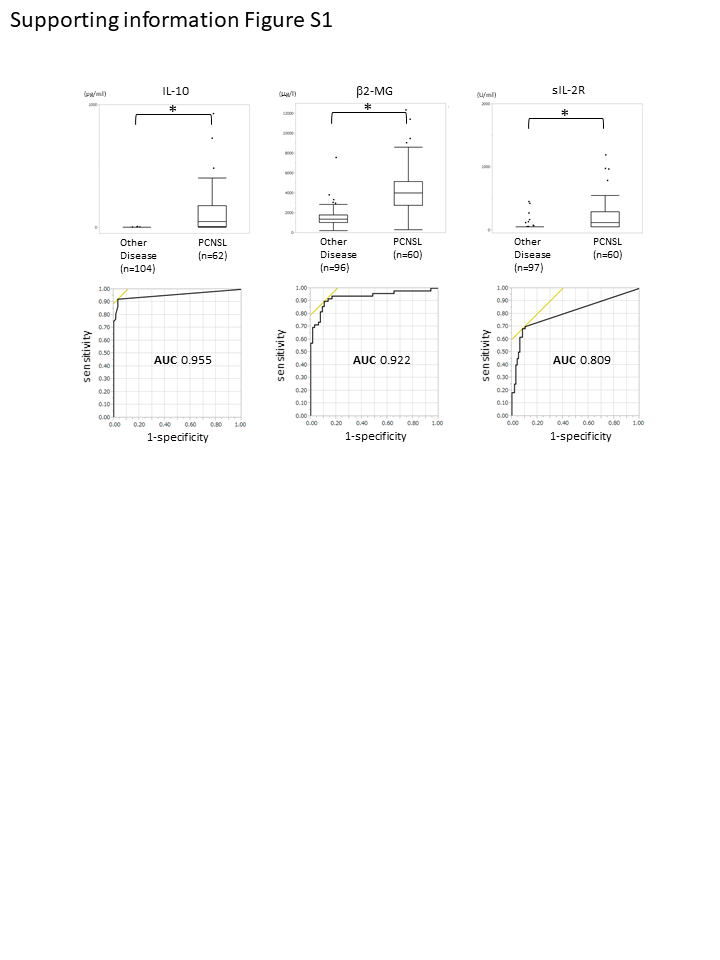

Supplement: Supplementary file 1 — Figure S1 [file CAM4-9-4114-s001.TIF]
