# Supplementary material for: Multi‐marker algorithms based on CXCL13, IL‐10, sIL‐2 receptor, and β2‐microglobulin in cerebrospinal fluid to diagnose CNS lymphoma
Source: Cancer Med. 2020 Apr 20;9(12):4114–25. doi: 10.1002/cam4.3048 (PMC7300423; doi:10.1002/cam4.3048)
Supplement: Supplementary file 2 — Table S1 [file CAM4-9-4114-s002.docx]

Supporting information Table S1. CSF data of all case-control study patients

| No. | case/control | category | pathology | CXCL13 (pg/ml) | IL-10 (pg/ml) | β2-MG (μg/l) | sIL-2R (U/ml) |
| --- | --- | --- | --- | --- | --- | --- | --- |
| 1 | case | PCNSL | PCNSL | 1543 | 1160 | 6620 | 6480 |
| 2 | case | PCNSL | PCNSL | 1536 | 13 | 4524 | 3495 |
| 3 | case | PCNSL | PCNSL | 1443 | 301 | 4249 | 2015 |
| 4 | case | PCNSL | PCNSL (recurrence) | 1361 | 17 | 11437 | 1192 |
| 5 | case | PCNSL | PCNSL | 1548 | 42 | 4560 | 978 |
| 6 | case | PCNSL | PCNSL | 324 | 35 | 3858 | 970 |
| 7 | case | PCNSL | PCNSL | 914 | 366 | 4338 | 784 |
| 8 | case | PCNSL | PCNSL | 1890 | 729 | 4018 | 546 |
| 9 | case | PCNSL | PCNSL | 1021 | 92 | 2704 | 483 |
| 10 | case | PCNSL | PCNSL (recurrence) | 1236 | 1040 | 12352 | 466 |
| 11 | case | PCNSL | PCNSL | 1625 | 69 | 4401 | 456 |
| 12 | case | PCNSL | PCNSL | 1493 | 930 | 8619 | 371 |
| 13 | case | PCNSL | PCNSL (recurrence) | 1880 | 1340 | 1186 | 361 |
| 14 | case | PCNSL | PCNSL | 219 | 51 | 8022 | 337 |
| 15 | case | PCNSL | PCNSL | 1527 | 40 | 3991 | 299 |
| 16 | case | PCNSL | PCNSL | 1780 | 1910 | 5500 | 256 |
| 17 | case | PCNSL | PCNSL | 3216 | 53 | 5279 | 233 |
| 18 | case | PCNSL | PCNSL | 1070 | 34 | 3361 | 227 |
| 19 | case | PCNSL | PCNSL | 1349 | 156 | 5027 | 222 |
| 20 | case | PCNSL | PCNSL | 1758 | 2 | 2787 | 194 |
| 21 | case | PCNSL | PCNSL | 1557 | 10 | 5200 | 191 |
| 22 | case | PCNSL | PCNSL | 1116 | 127 | 4490 | 189 |
| 23 | case | PCNSL | PCNSL | 1411 | 483 | 6697 | 179 |
| 24 | case | PCNSL | PCNSL | 1415 | 88 | 2339 | 167 |
| 25 | case | PCNSL | PCNSL | 1026 | 41 | 5805 | 160 |
| 26 | case | PCNSL | PCNSL | 1267 | 1090 | 9500 | 141 |
| 27 | case | PCNSL | PCNSL | 1700 | 39 | 2917 | 136 |
| 28 | case | PCNSL | PCNSL | 1479 | 231 | 3077 | 122 |
| 29 | case | PCNSL | PCNSL | 1625 | 231 | 3077 | 122 |
| 30 | case | PCNSL | PCNSL | 564 | 51 | 3343 | 120 |
| 31 | case | PCNSL | PCNSL | 1782 | 160 | 7460 | 115 |
| 32 | case | PCNSL | PCNSL | 1371 | 29 | 2074 | 101 |
| 33 | case | PCNSL | PCNSL | 1694 | 47 | 1209 | 89 |
| 34 | case | PCNSL | PCNSL | 1254 | 118 | 6137 | 88 |
| 35 | case | PCNSL | PCNSL | 1521 | 63 | 9071 | 85 |
| 36 | case | PCNSL | PCNSL | 1535 | 3 | 5066 | 78 |
| 37 | case | PCNSL | PCNSL | 517 | 147 | 4187 | 67 |
| 38 | case | PCNSL | PCNSL | 1579 | 42 | 4983 | 62 |
| 39 | case | PCNSL | PCNSL | 2904 | 62 | 4693 | 60 |
| 40 | case | PCNSL | PCNSL | 1020 | 29 | 4830 | 60 |
| 41 | case | PCNSL | PCNSL | 1590 | 8 | 2812 | 51 |
| 42 | case | PCNSL | PCNSL | 1294 | 52 | 4532 | 50 |
| 43 | case | PCNSL | PCNSL | 1104 | 5 | 3854 | 50 |
| 44 | case | PCNSL | PCNSL | 1004 | 6 | 3543 | 50 |
| 45 | case | PCNSL | PCNSL | 1897 | 404 | 3426 | 50 |
| 46 | case | PCNSL | PCNSL | 1649 | 379 | 3024 | 50 |
| 47 | case | PCNSL | PCNSL | 733 | 54 | 2753 | 50 |
| 48 | case | PCNSL | PCNSL | 436 | 5 | 2703 | 50 |
| 49 | case | PCNSL | PCNSL | 1935 | 5 | 2303 | 50 |
| 50 | case | PCNSL | PCNSL | 670 | 2 | 2260 | 50 |
| 51 | case | PCNSL | PCNSL | 335 | 4 | 2256 | 50 |
| 52 | case | PCNSL | PCNSL | 1317 | 48 | 1916 | 50 |
| 53 | case | PCNSL | PCNSL | 400 | 7 | 1080 | 50 |
| 54 | case | PCNSL | PCNSL | 1506 | 2 | 317 | 50 |
| 55 | case | PCNSL | PCNSL | 1648 | 5940 | - | - |
| 56 | case | PCNSL | PCNSL | 1333 | 4 | - | - |
| 57 | case | PCNSL | PCNSL | 680 | - | - | - |
| 58 | case | PCNSL | PCNSL | 1471 | - | - | - |
| 59 | case | PCNSL | PCNSL | 1589 | - | - | - |
| 60 | case | PCNSL | PCNSL (recurrence) | 1606 | - | - | - |
| 61 | case | PCNSL | PCNSL | 1797 | - | - | - |
| 62 | case | PCNSL | PCNSL | 2614 | - | - | - |
| 63 | case | PCNSL | PCNSL | 2833 | - | - | - |
| 64 | case | PCNSL | PCNSL | 4888 | - | - | - |
| 65 | case | PCNSL | PCNSL | 2962 | - | - | - |
| 66 | case | PCNSL | PCNSL | 1543 | - | - | - |
| 67 | control | glioblastoma | glioblastoma | 1 | 2 | 1037 | 50 |
| 68 | control | glioblastoma | glioblastoma | 1 | 2 | 1327 | 50 |
| 69 | control | glioblastoma | glioblastoma | 1 | 2 | 1401 | 50 |
| 70 | control | glioblastoma | glioblastoma | 1 | 2 | 1537 | 50 |
| 71 | control | glioblastoma | glioblastoma | 1 | 2 | 1781 | 50 |
| 72 | control | glioblastoma | glioblastoma | 1 | 2 | 3074 | 50 |
| 73 | control | glioblastoma | glioblastoma | 1 | 2 | 1326 | 50 |
| 74 | control | glioblastoma | glioblastoma | 1 | 2 | 1612 | 50 |
| 75 | control | glioblastoma | glioblastoma | 1 | 2 | 330 | 50 |
| 76 | control | glioblastoma | glioblastoma | 4 | 2 | 1300 | 50 |
| 77 | control | glioblastoma | glioblastoma | 4 | 2 | 1899 | 50 |
| 78 | control | glioblastoma | glioblastoma | 4 | 2 | 1202 | 50 |
| 79 | control | glioblastoma | glioblastoma | 5 | 2 | 1043 | 50 |
| 80 | control | glioblastoma | glioblastoma | 5 | 2 | 1776 | 50 |
| 81 | control | glioblastoma | glioblastoma | 6 | 2 | 1109 | 50 |
| 82 | control | glioblastoma | glioblastoma | 6 | 5 | 1506 | 50 |
| 83 | control | glioblastoma | glioblastoma | 6 | 2 | 3324 | 50 |
| 84 | control | glioblastoma | glioblastoma | 6 | 2 | 244 | 50 |
| 85 | control | glioblastoma | glioblastoma | 8 | 2 | 1689 | 50 |
| 86 | control | glioblastoma | glioblastoma | 8 | 2 | 2804 | 50 |
| 87 | control | glioblastoma | glioblastoma | 10 | 2 | 2279 | 50 |
| 88 | control | glioblastoma | glioblastoma | 11 | 2 | 2158 | 50 |
| 89 | control | glioblastoma | glioblastoma | 12 | 2 | 1604 | 50 |
| 90 | control | glioblastoma | glioblastoma | 12 | 2 | 2055 | 50 |
| 91 | control | glioblastoma | glioblastoma | 13 | 2 | 1756 | 50 |
| 92 | control | glioblastoma | glioblastoma | 14 | 2 | 1368 | 50 |
| 93 | control | glioblastoma | glioblastoma | 14 | 2 | 1698 | 50 |
| 94 | control | glioblastoma | glioblastoma | 16 | 2 | 1021 | 50 |
| 95 | control | glioblastoma | glioblastoma | 17 | 2 | 1168 | 50 |
| 96 | control | glioblastoma | glioblastoma | 18 | 2 | 1682 | 50 |
| 97 | control | glioblastoma | glioblastoma | 22 | 2 | 1646 | 50 |
| 98 | control | glioblastoma | glioblastoma | 23 | 2 | 745 | 50 |
| 99 | control | glioblastoma | glioblastoma | 24 | 2 | 556 | 50 |
| 100 | control | glioblastoma | glioblastoma | 67 | 2 | 1150 | 50 |
| 101 | control | glioblastoma | glioblastoma | 68 | 2 | 2984 | 50 |
| 102 | control | glioblastoma | glioblastoma | 249 | 2 | 1278 | 50 |
| 103 | control | glioblastoma | glioblastoma | 7 | 2 | - | - |
| 104 | control | glioblastoma | glioblastoma | 1 | - | - | - |
| 105 | control | glioblastoma | glioblastoma | 1 | - | - | - |
| 106 | control | glioblastoma | glioblastoma | 1 | - | - | - |
| 107 | control | glioblastoma | glioblastoma | 2 | - | - | - |
| 108 | control | glioblastoma | glioblastoma | 4 | - | - | - |
| 109 | control | glioblastoma | glioblastoma | 4 | - | - | - |
| 110 | control | glioblastoma | glioblastoma | 5 | - | - | - |
| 111 | control | glioblastoma | glioblastoma | 5 | - | - | - |
| 112 | control | glioblastoma | glioblastoma | 6 | - | - | - |
| 113 | control | glioblastoma | glioblastoma | 7 | - | - | - |
| 114 | control | glioblastoma | glioblastoma | 7 | - | - | - |
| 115 | control | glioblastoma | glioblastoma | 8 | - | - | - |
| 116 | control | glioblastoma | glioblastoma | 11 | - | - | - |
| 117 | control | glioblastoma | glioblastoma | 15 | - | - | - |
| 118 | control | glioblastoma | glioblastoma | 15 | - | - | - |
| 119 | control | glioblastoma | glioblastoma | 18 | - | - | - |
| 120 | control | glioblastoma | glioblastoma | 19 | - | - | - |
| 121 | control | glioblastoma | glioblastoma | 25 | - | - | - |
| 122 | control | glioblastoma | glioblastoma | 25 | - | - | - |
| 123 | control | glioblastoma | glioblastoma | 28 | - | - | - |
| 124 | control | glioblastoma | glioblastoma | 55 | - | - | - |
| 125 | control | glioblastoma | glioblastoma | 345 | - | - | - |
| 126 | control | other glioma | anaplastic astrocytoma | 86 | 2 | - | 50 |
| 127 | control | other glioma | anaplastic astrocytoma | 8 | 2 | 842 | 50 |
| 128 | control | other glioma | anaplastic astrocytoma | 15 | 2 | 1010 | 50 |
| 129 | control | other glioma | anaplastic astrocytoma | 13 | 2 | 1068 | 50 |
| 130 | control | other glioma | anaplastic astrocytoma | 1 | 2 | 1509 | 50 |
| 131 | control | other glioma | anaplastic astrocytoma | 19 | 2 | 1517 | 50 |
| 132 | control | other glioma | anaplastic astrocytoma | 20 | 2 | 1685 | 50 |
| 133 | control | other glioma | diffuse astrocytoma | 13 | 2 | 300 | 50 |
| 134 | control | other glioma | diffuse astrocytoma | 5 | 2 | 728 | 50 |
| 135 | control | other glioma | diffuse astrocytoma | 23 | 2 | 800 | 50 |
| 136 | control | other glioma | diffuse astrocytoma | 3 | 2 | 1374 | 50 |
| 137 | control | other glioma | diffuse astrocytoma | 275 | 2 | 1439 | 50 |
| 138 | control | other glioma | diffuse astrocytoma | 4 | 2 | 1971 | 50 |
| 139 | control | other glioma | diffuse astrocytoma | 13 | 2 | 1172 | 421 |
| 140 | control | other glioma | ependymoma | 37 | 2 | 200 | 50 |
| 141 | control | other glioma | ependymoma | 35 | 2 | 1000 | 50 |
| 142 | control | other glioma | oligodendroglioma | 8 | 2 | 1534 | 50 |
| 143 | control | other glioma | oligodendroglioma | 14 | 2 | 1563 | 50 |
| 144 | control | other glioma | oligodendroglioma | 14 | 2 | 2075 | 50 |
| 145 | control | other glioma | pontine glioma | 4 | 2 | 913 | 50 |
| 146 | control | other glioma | pontine glioma | 4 | 2 | 1403 | 50 |
| 147 | control | other glioma | anaplastic astrocytoma | 12 | 2 | - | 50 |
| 148 | control | other glioma | anaplastic astrocytoma | 14 | 2 | - | 50 |
| 149 | control | other glioma | anaplastic astrocytoma | 4 | - | - | - |
| 150 | control | other glioma | anaplastic astrocytoma | 6 | - | - | - |
| 151 | control | other glioma | anaplastic astrocytoma | 54 | - | - | - |
| 152 | control | other glioma | anaplastic astrocytoma | 55 | - | - | - |
| 153 | control | other glioma | anaplastic ependymoma | 7 | - | - | - |
| 154 | control | other glioma | anaplastic oligoastrocytoma | 12 | - | - | - |
| 155 | control | other glioma | diffuse astrocytoma | 8 | - | - | - |
| 156 | control | other glioma | diffuse astrocytoma | 10 | - | - | - |
| 157 | control | other glioma | diffuse astrocytoma | 11 | - | - | - |
| 158 | control | other glioma | diffuse astrocytoma | 25 | - | - | - |
| 159 | control | other glioma | oligodendroglioma | 17 | - | - | - |
| 160 | control | other glioma | pilocytic astrocytoma | 20 | 2 | 878 | 50 |
| 161 | control | other glioma | pilocytic astrocytoma | 4 | - | - | - |
| 162 | control | germ cell tumor | germinoma | 6 | 2 | 200 | 50 |
| 163 | control | germ cell tumor | germinoma | 8 | 2 | 627 | 50 |
| 164 | control | germ cell tumor | germinoma | 15 | 2 | 1163 | 50 |
| 165 | control | germ cell tumor | teratoma | 82 | 2 | 1200 | 55 |
| 166 | control | germ cell tumor | germinoma | 76 | 2 | - | 50 |
| 167 | control | germ cell tumor | germinoma | 1 | - | - | - |
| 168 | control | germ cell tumor | germinoma | 4 | - | - | - |
| 169 | control | germ cell tumor | germinoma | 7 | - | - | - |
| 170 | control | germ cell tumor | germinoma | 15 | - | - | - |
| 171 | control | germ cell tumor | germinoma | 23 | - | - | - |
| 172 | control | germ cell tumor | germinoma | 23 | - | - | - |
| 173 | control | germ cell tumor | germinoma | 334 | - | - | - |
| 174 | control | germ cell tumor | teratoma | 1 | - | - | - |
| 175 | control | germ cell tumor | teratoma | 6 | - | - | - |
| 176 | control | germ cell tumor | teratoma | 13 | - | - | - |
| 177 | control | germ cell tumor | teratoma | 24 | - | - | - |
| 178 | control | germ cell tumor | Yolk sac tumor | 7 | - | - | - |
| 179 | control | metastatic brain tumor | metastatic brain tumor | 6 | 2 | 1116 | 50 |
| 180 | control | metastatic brain tumor | metastatic brain tumor | 7 | 2 | 1800 | 131 |
| 181 | control | metastatic brain tumor | metastatic brain tumor | 12 | 2 | 3813 | 450 |
| 182 | control | metastatic brain tumor | metastatic brain tumor | 74 | 2 | 2392 | 76 |
| 183 | control | metastatic brain tumor | metastatic brain tumor | 84 | 2 | 942 | 50 |
| 184 | control | metastatic brain tumor | metastatic brain tumor | 664 | 2 | 1925 | 117 |
| 185 | control | metastatic brain tumor | metastatic brain tumor | 1391 | 2 | 1039 | 50 |
| 186 | control | metastatic brain tumor | metastatic brain tumor | 9 | 2 | - | 50 |
| 187 | control | metastatic brain tumor | metastatic brain tumor | 6 | 2 | - | 53 |
| 188 | control | metastatic brain tumor | metastatic brain tumor | 1 | 2 | - |  |
| 189 | control | metastatic brain tumor | metastatic brain tumor | 4 | - | - | - |
| 190 | control | metastatic brain tumor | metastatic brain tumor | 6 | - | - | - |
| 191 | control | metastatic brain tumor | metastatic brain tumor | 26 | - | - | - |
| 192 | control | other tumor | ATLL | 9 | 4 | 1981 | 166 |
| 193 | control | other tumor | chordoma | 5 | 2 | 932 | 50 |
| 194 | control | other tumor | chordoma | 6 | 2 | 2396 | 50 |
| 195 | control | other tumor | medullobalstoma | 1 | 2 | 708 | 50 |
| 196 | control | other tumor | meningioma | 4 | 2 | 1164 | 50 |
| 197 | control | other tumor | cavernous angioma | 7 | 2 | 966 | 50 |
| 198 | control | other tumor | cavernous angioma | 1 | - | - | - |
| 199 | control | other tumor | central neurocytoma | 1 | - | - | - |
| 200 | control | other tumor | craniopharyngioma | 6 | - | - | - |
| 201 | control | other tumor | glioneuronal tumor | 5 | - | - | - |
| 202 | control | other tumor | granuloma | 6 | - | - | - |
| 203 | control | other tumor | hemangioblastoma | 1 | - | - | - |
| 204 | control | other tumor | meningioma | 7 | - | - | - |
| 205 | control | other tumor | temporal cyst | 5 | - | - | - |
| 206 | control | multiple sclerosis | multiple sclerosis | 1 | 2 | 1006 | 50 |
| 207 | control | multiple sclerosis | multiple sclerosis | 5 | 2 | 1344 | 50 |
| 208 | control | multiple sclerosis | multiple sclerosis | 5 | 2 | 1804 | 50 |
| 209 | control | multiple sclerosis | multiple sclerosis | 7 | 2 | 1348 | 50 |
| 210 | control | multiple sclerosis | multiple sclerosis | 8 | 2 | 1115 | 50 |
| 211 | control | multiple sclerosis | multiple sclerosis | 8 | 2 | 862 | 50 |
| 212 | control | multiple sclerosis | multiple sclerosis | 9 | 2 | 1249 | 50 |
| 213 | control | multiple sclerosis | multiple sclerosis | 25 | 2 | 1784 | 50 |
| 214 | control | multiple sclerosis | multiple sclerosis | 28 | 2 | 2111 | 50 |
| 215 | control | multiple sclerosis | multiple sclerosis | 1 | 2 | 859 | 50 |
| 216 | control | multiple sclerosis | multiple sclerosis | 85 | 2 | - | - |
| 217 | control | multiple sclerosis | multiple sclerosis | 6 | - | - | - |
| 218 | control | multiple sclerosis | multiple sclerosis | 24 | - | - | - |
| 219 | control | multiple sclerosis | multiple sclerosis | 287 | - | - | - |
| 220 | control | iNPH | iNPH | 3 | - | - | - |
| 221 | control | iNPH | iNPH | 4 | - | - | - |
| 222 | control | iNPH | iNPH | 1 | - | - | - |
| 223 | control | iNPH | iNPH | 1 | - | - | - |
| 224 | control | iNPH | iNPH | 4 | - | - | - |
| 225 | control | iNPH | iNPH | 4 | - | - | - |
| 226 | control | iNPH | iNPH | 4 | - | - | - |
| 227 | control | iNPH | iNPH | 6 | - | - | - |
| 228 | control | iNPH | iNPH | 6 | - | - | - |
| 229 | control | iNPH | iNPH | 109 | - | - | - |
| 230 | control | other disease | headache | 4 | 2 | 1109 | 50 |
| 231 | control | other disease | IgG4-related disease | 1569 | 2 | 1921 | 70 |
| 232 | control | other disease | leukoencephalopthy | 23 | 2 | 1076 | 50 |
| 233 | control | other disease | meningitis | 23 | 2 | 2845 | 50 |
| 234 | control | other disease | sarcoidosis | 1452 | 2 | 1100 | 50 |
| 235 | control | other disease | abscess | 36 | - | - | - |
| 236 | control | other disease | headache | 1 | - | - | - |
| 237 | control | other disease | headache | 4 | - | - | - |
| 238 | control | other disease | headache | 4 | - | - | - |
| 239 | control | other disease | headache | 5 | - | - | - |
| 240 | control | other disease | headache | 6 | - | - | - |
| 241 | control | other disease | headache | 18 | - | - | - |
| 242 | control | other disease | hematoma | 7 | - | - | - |
| 243 | control | other disease | IgG4-related disease | 1044 | - | - | - |
| 244 | control | other disease | meningitis | 6 | - | - | - |
| 245 | control | other disease | sarcoidosis | 6 | - | - | - |
| 246 | control | other disease | sarcoidosis | 7 | - | - | - |
| 247 | control | other disease | sarcoidosis | 21 | - | - | - |
| 248 | control | other disease | venous thrombosis | 4 | - | - | - |
