# Supplementary material for: Multi‐marker algorithms based on CXCL13, IL‐10, sIL‐2 receptor, and β2‐microglobulin in cerebrospinal fluid to diagnose CNS lymphoma
Source: Cancer Med. 2020 Apr 20;9(12):4114–25. doi: 10.1002/cam4.3048 (PMC7300423; doi:10.1002/cam4.3048)
Supplement: Supplementary file 3 — Table S2 [file CAM4-9-4114-s003.docx]

Supporting information Table S2. The data of the patients of the prospective study

| No. | Age | Sex | Pathology | CXCL13  (pg/ml) | IL-10  (pg/ml) | β2MG (μg/l) | sIL2R  (U/ml) | Probability  (CXCL13, IL-10,  β2-MG  sIL-2R) | Probability  (CXCL13, IL-10,  β2-MG) | Probability  (CXCL13, IL-10,  sIL-2R) | Probability  (CXCL13, IL-10) |
| --- | --- | --- | --- | --- | --- | --- | --- | --- | --- | --- | --- |
| 1 | 87 | F | GBM | 11 | 2 | 2158 | 50 | 0.01 | 0.01 | 0.00 | 0.00 |
| 2 | 21 | M | Pontine glioma | 4 | 2 | 1403 | 50 | 0.00 | 0.00 | 0.00 | 0.00 |
| 3 | 60 | F | PCNSL (rec) | 914 | 48 | 2791 | 50 | 1.00 | 1.00 | 1.00 | 1.00 |
| 4 | 74 | M | GBM | 4 | 2 | 1899 | 50 | 0.01 | 0.01 | 0.00 | 0.00 |
| 5 | 50 | F | Multiple sclerosis | 5 | 2 | 1344 | 50 | 0.00 | 0.00 | 0.00 | 0.00 |
| 6 | 72 | M | PCNSL (rec) | 1880 | 440 | 4479 | 78 | 1.00 | 1.00 | 1.00 | 1.00 |
| 7 | 84 | M | GBM | 6 | 2 | 3324 | 50 | 0.04 | 0.03 | 0.00 | 0.00 |
| 8 | 59 | F | ATLL | 9 | 4 | 1981 | 166 | 0.12 | 0.20 | 0.23 | 0.21 |
| 9 | 71 | M | PCNSL | 1700 | 39 | 2917 | 136 | 1.00 | 1.00 | 1.00 | 1.00 |
| 10 | 64 | F | Multiple sclerosis | 8 | 2 | 1115 | 50 | 0.00 | 0.00 | 0.00 | 0.00 |
| 11 | 24 | M | Pontine glioma | 4 | 2 | 913 | 50 | 0.00 | 0.00 | 0.00 | 0.00 |
| 12 | 71 | F | GBM | 7 | 2 | 1300 | 50 | 0.00 | 0.00 | 0.00 | 0.00 |
| 13 | 31 | F | Multiple sclerosis | 6 | 2 | 1348 | 50 | 0.00 | 0.00 | 0.00 | 0.00 |
| 14 | 78 | M | PCNSL | 1401 | 34 | 3551 | 103 | 1.00 | 1.00 | 1.00 | 1.00 |
| 16 | 30 | M | Germinoma | 1 | 2 | 1122 | 50 | 0.00 | 0.00 | 0.00 | 0.00 |
| 15 | 41 | F | Multiple sclerosis | 10 | 2 | 1473 | 50 | 0.00 | 0.00 | 0.00 | 0.00 |
| 17 | 69 | M | GBM | 4 | 2 | 1591 | 50 | 0.00 | 0.00 | 0.00 | 0.00 |
| 18 | 77 | M | GBM | 9 | 2 | 2808 | 50 | 0.02 | 0.02 | 0.00 | 0.00 |
| 19 | 74 | F | Intravascular lymphoma | 660 | 13 | 3154 | 311 | 1.00 | 1.00 | 1.00 | 1.00 |
| 20 | 33 | M | Mdulloblastoma | 8 | 2 | 1209 | 50 | 0.00 | 0.00 | 0.00 | 0.00 |
| 21 | 84 | M | Leukoencephalopathy | 3 | 2 | 1199 | 50 | 0.00 | 0.00 | 0.00 | 0.00 |
| 22 | 73 | M | PCNSL | 2228 | 27 | 3554 | 81 | 1.00 | 1.00 | 1.00 | 1.00 |
| 25 | 78 | M | PCNSL | 2137 | 46 | 3497 | 116 | 1.00 | 1.00 | 1.00 | 1.00 |
| 23 | 56 | M | GBM | 2 | 2 | 1353 | 50 | 0.00 | 0.00 | 0.00 | 0.00 |
| 24 | 76 | F | Astrocytoma | 8 | 4 | 1257 | 50 | 0.09 | 0.09 | 0.19 | 0.21 |
| 26 | 79 | M | PCNSL | 10 | 40 | 3711 | 61 | 1.00 | 1.00 | 1.00 | 1.00 |
| 27 | 79 | F | PCNSL | 1804 | 1240 | 4062 | 793 | 1.00 | 1.00 | 1.00 | 1.00 |
| 28 | 55 | F | Glioma | 12 | 2 | 1761 | 50 | 0.00 | 0.00 | 0.00 | 0.00 |
| 29 | 75 | M | Lymphomatosis | 59 | 2 | 2313 | 50 | 0.01 | 0.01 | 0.00 | 0.01 |
| 30 | 76 | M | Venous thrombosis | 9 | 2 | 1400 | 50 | 0.00 | 0.00 | 0.00 | 0.00 |
| 31 | 62 | F | Glioma | 14 | 2 | 1552 | 50 | 0.00 | 0.00 | 0.00 | 0.00 |
| 32 | 24 | F | Glioma | 4 | 2 | 1118 | 50 | 0.00 | 0.00 | 0.00 | 0.00 |
| 33 | 53 | M | GBM | 7 | 2 | 1590 | 50 | 0.00 | 0.00 | 0.00 | 0.00 |
| 34 | 77 | M | Leukoencephalopathy | 49 | 2 | 2700 | 50 | 0.02 | 0.02 | 0.00 | 0.00 |
| 35 | 58 | M | SCNSL | 1808 | 156 | 4405 | 323 | 1.00 | 1.00 | 1.00 | 1.00 |
| 36 | 40 | M | GBM | 38 | 2 | 1423 | 50 | 0.00 | 0.00 | 0.00 | 0.00 |
| 37 | 48 | M | Meningioma | 9 | 2 | 1123 | 50 | 0.00 | 0.00 | 0.00 | 0.00 |
| 38 | 78 | F | PCNSL | 1013 | 22 | 4314 | 69 | 1.00 | 1.00 | 1.00 | 1.00 |
| 39 | 20 | M | Germinoma | 269 | 2 | 1817 | 50 | 0.01 | 0.01 | 0.01 | 0.01 |
| 40 | 39 | F | Anaplastic ependymoma | 229 | 2 | 1643 | 50 | 0.01 | 0.01 | 0.01 | 0.01 |
| 42 | 39 | M | Astrocytoma | 1 | 2 | 1028 | 50 | 0.00 | 0.00 | 0.00 | 0.00 |
| 41 | 81 | F | Metastatic tumor (Bladdar ca.） | 2 | 2 | 1439 | 50 | 0.00 | 0.00 | 0.00 | 0.00 |
| 43 | 42 | M | Abscess | 5 | 2 | 1371 | 50 | 0.00 | 0.00 | 0.00 | 0.00 |
| 44 | 56 | F | PCNSL | 1717 | 15 | 4031 | 101 | 1.00 | 1.00 | 1.00 | 1.00 |
| 45 | 65 | F | PCNSL | 447 | 5 | 2319 | 50 | 0.94 | 0.93 | 0.91 | 0.91 |
| 46 | 66 | F | SCNSL | 1559 | 111 | 4287 | 150 | 1.00 | 1.00 | 1.00 | 1.00 |
| 47 | 39 | M | Subspendymoma | 14 | 2 | 1000 | 50 | 0.00 | 0.00 | 0.00 | 0.00 |
| 48 | 78 | M | PCNSL | 1543 | 77 | 6300 | 3740 | 1.00 | 1.00 | 1.00 | 1.00 |
| 49 | 63 | F | GBM | 1 | 2 | 1531 | 50 | 0.00 | 0.00 | 0.00 | 0.00 |
| 50 | 46 | F | PCNSL | 1128 | 4 | 1972 | 280 | 0.86 | 0.95 | 0.96 | 0.94 |
| 51 | 24 | M | Abscess | 16 | 2 | 1200 | 50 | 0.00 | 0.00 | 0.00 | 0.00 |
| 52 | 33 | M | Cortical dysplasia | 1 | 2 | 931 | 50 | 0.00 | 0.00 | 0.00 | 0.00 |
| 53 | 40 | M | Metastatic tumor (Lung ca.) | 1 | 2 | 1121 | 50 | 0.00 | 0.00 | 0.00 | 0.00 |
| 54 | 77 | M | IOL（PCNSL) | 553 | 13 | 2750 | 50 | 1.00 | 1.00 | 1.00 | 1.00 |
| 55 | 68 | F | PCNSL | 2134 | 71 | 6112 | 734 | 1.00 | 1.00 | 1.00 | 1.00 |
| 56 | 42 | F | Meningitis | 251 | 2 | 1506 | 50 | 0.01 | 0.01 | 0.01 | 0.01 |
| 57 | 69 | F | GBM | 11 | 2 | 1503 | 50 | 0.00 | 0.00 | 0.00 | 0.00 |
| 61 | 78 | M | PCNSL | 684 | 11 | 1700 | 50 | 1.00 | 1.00 | 1.00 | 1.00 |
| 60 | 39 | M | Astrocytoma | 1 | 2 | 1462 | 50 | 0.00 | 0.00 | 0.00 | 0.00 |
| 58 | 83 | M | GBM | 1 | 2 | 1252 | 50 | 0.00 | 0.00 | 0.00 | 0.00 |
| 59 | 30 | M | Oligodendroglioma | 1 | 2 | 793 | 50 | 0.00 | 0.00 | 0.00 | 0.00 |
| 62 | 83 | M | PCNSL | 1690 | 31 | 6545 | 1438 | 1.00 | 1.00 | 1.00 | 1.00 |
| 63 | 54 | F | Sjögren's syndrome | 874 | 2 | 3877 | 55 | 0.73 | 0.62 | 0.09 | 0.09 |
| 64 | 47 | F | Multiple sclerosis | 22 | 2 | 1366 | 50 | 0.00 | 0.00 | 0.00 | 0.00 |
| 65 | 40 | M | Diffuse midline glioma | 25 | 2 | 2063 | 50 | 0.01 | 0.01 | 0.00 | 0.00 |
| 66 | 63 | M | PCNSL | 1952 | 2 | 2240 | 133 | 0.93 | 0.94 | 0.86 | 0.85 |
| 67 | 62 | M | Infarctrion | 24 | 2 | 1280 | 50 | 0.00 | 0.00 | 0.00 | 0.00 |
| 69 | 60 | F | PCNSL | 1525 | 17 | 4763 | 150 | 1.00 | 1.00 | 1.00 | 1.00 |
| 68 | 16 | F | GBM | 5 | 2 | 200 | 50 | 0.00 | 0.00 | 0.00 | 0.00 |
| 70 | 77 | F | GBM | 1 | 2 | 1721 | 50 | 0.00 | 0.00 | 0.00 | 0.00 |
| 71 | 32 | M | Diffuse midline glioma | 32 | 2 | 2380 | 59 | 0.01 | 0.01 | 0.00 | 0.00 |
| 72 | 50 | F | GBM | 17 | 2 | 1235 | 50 | 0.00 | 0.00 | 0.00 | 0.00 |
| 73 | 82 | F | GBM | 3 | 2 | 595 | 50 | 0.00 | 0.00 | 0.00 | 0.00 |
| 74 | 71 | M | B-lymphoblastic leukemia/lymphoma | 1543 | 9 | 3495 | 736 | 1.00 | 1.00 | 1.00 | 1.00 |
| 75 | 50 | F | GBM | 16 | 2 | 1649 | 50 | 0.00 | 0.00 | 0.00 | 0.00 |
| 76 | 18 | F | Astrocytoma | 1 | 2 | 743 | 50 | 0.00 | 0.00 | 0.00 | 0.00 |
| 77 | 61 | F | Metastatic tumor (Colon ca.) | 30 | 2 | 1023 | 50 | 0.00 | 0.00 | 0.00 | 0.00 |
| 78 | 40 | M | Pontine glioma | 77 | 2 | 929 | 50 | 0.00 | 0.00 | 0.01 | 0.01 |
| 79 | 51 | M | Oligodendroglioma | 107 | 2 | 1283 | 50 | 0.00 | 0.00 | 0.01 | 0.01 |
| 80 | 64 | M | GBM | 1267 | 2 | 2648 | 50 | 0.70 | 0.64 | 0.30 | 0.31 |
| 81 | 70 | F | PCNSL | 3231 | 49 | 9328 | 297 | 1.00 | 1.00 | 1.00 | 1.00 |
| 82 | 70 | M | PCNSL | 2863 | 2 | 7810 | 175 | 1.00 | 1.00 | 0.99 | 0.99 |
| 83 | 44 | F | Histiocytic sarcoma | 2153 | 2 | 5651 | 320 | 1.00 | 1.00 | 0.95 | 0.92 |
| 84 | 79 | F | Anaplastic astrocytoma | 62 | 2 | 2272 | 50 | 0.01 | 0.01 | 0.00 | 0.01 |
| 85 | 56 | F | Metastatic tumor (Brest) | 1910 | 6 | 2313 | 92 | 1.00 | 1.00 | 1.00 | 1.00 |
| 86 | 66 | M | PCNSL | 4116 | 10 | 4127 | 3009 | 1.00 | 1.00 | 1.00 | 1.00 |
| 87 | 78 | F | PCNSL | 221 | 69 | 1800 | 86.2 | 1.00 | 1.00 | 1.00 | 1.00 |
| 88 | 23 | F | GBM | 1 | 2 | 806 | 50 | 0.00 | 0.00 | 0.00 | 0.00 |
| 89 | 76 | F | PCNSL | 3858 | 433 | 5000 | 191 | 1.00 | 1.00 | 1.00 | 1.00 |
| 90 | 39 | F | Diffuse astrocytoma | 1 | 3 | 1268 | 50 | 0.01 | 0.02 | 0.03 | 0.03 |
| 91 | 58 | F | GBM | 1 | 2 | 1841 | 50 | 0.00 | 0.00 | 0.00 | 0.00 |
| 92 | 23 | F | Cortical dysplasia | 5 | 2 | 883 | 50 | 0.00 | 0.00 | 0.00 | 0.00 |
| 93 | 33 | M | Hemangioblastoma | 1 | 2 | 400 | 50 | 0.00 | 0.00 | 0.00 | 0.00 |
| 94 | 48 | F | Meningioma | 3 | 2 | 191 | 50 | 0.00 | 0.00 | 0.00 | 0.00 |
| 95 | 16 | F | Hydrocephalus | 1 | 2 | 619 | 50 | 0.00 | 0.00 | 0.00 | 0.00 |
| 96 | 16 | M | Yolk sac tumor | 345 | 4 | 850 | 50 | 0.17 | 0.19 | 0.46 | 0.48 |
| 97 | 68 | F | Metastatic tumor (Lung ca.) | 1 | 2 | 1908 | 50 | 0.01 | 0.01 | 0.00 | 0.00 |
| 98 | 78 | M | PCNSL | 2633 | 198 | 3704 | 95 | 1.00 | 1.00 | 1.00 | 1.00 |
| 99 | 78 | F | PCNSL | 3388 | 16 | 4157 | 442 | 1.00 | 1.00 | 1.00 | 1.00 |
| 100 | 85 | F | Glioma | 2 | 2 | 2150 | 50 | 0.01 | 0.01 | 0.00 | 0.00 |
| 101 | 54 | F | PCNSL | 506 | 2 | 2219 | 50 | 0.06 | 0.05 | 0.00 | 0.03 |
| 102 | 67 | F | GBM | 29 | 2 | 2219 | 50 | 0.01 | 0.01 | 0.00 | 0.00 |
| 103 | 62 | M | SCNSL | 2607 | 34 | 3587 | 244 | 1.00 | 1.00 | 1.00 | 1.00 |
| 104 | 47 | M | Glioma | 3 | 2 | 1043 | 50 | 0.00 | 0.00 | 0.00 | 0.00 |
