# Supplementary material for: Multi‐marker algorithms based on CXCL13, IL‐10, sIL‐2 receptor, and β2‐microglobulin in cerebrospinal fluid to diagnose CNS lymphoma
Source: Cancer Med. 2020 Apr 20;9(12):4114–25. doi: 10.1002/cam4.3048 (PMC7300423; doi:10.1002/cam4.3048)
Supplement: Supplementary file 4 — Table S3 [file CAM4-9-4114-s004.docx]

Supporting information Table S3: 　Characteristic of PCNSL patients in survival analysis

|  | All PCNSL patients | High CSF CXCL13  (>1500 pg/ml) | Low CSF CXCL13  (<1500 pg/ml) | p-value |
| --- | --- | --- | --- | --- |
| Number of the patients | 63 | 32 | 31 | ― |
|  |  |  |  |  |
| Age mean | 64.5 | 66.4 | 62.5 |  |
|  |  |  |  |  |
| Sex |  |  |  | n.s. |
| men | 37 | 21 | 16 |  |
| women | 26 | 11 | 15 |  |
|  |  |  |  |  |
| KPS mean | 66.8 | 65.0 | 68.7 | n.s. |
|  |  |  |  |  |
| Treatment |  |  |  | 0.04 |
| RT + CT | 49 | 21 | 28 |  |
| RT only | 2 | 1 | 1 |  |
| CT only | 12 | 10 | 2 |  |
|  |  |  |  |  |
| Recurrence |  |  |  | n.s. |
| (-) | 26 | 13 | 13 |  |
| (+) | 37 | 19 | 18 |  |
|  |  |  |  |  |
| Status |  |  |  | n.s. |
| alive | 27 | 13 | 13 |  |
| dead | 37 | 19 | 18 |  |
|  |  |  |  |  |

PCNSL: primary CNS lymphoma, KPS: Karnofsky performance status, RT: radiotherapy,

CT: chemotherapy, n.s.: not significant
